# Phase I/II clinical trial of adoptive cell transfer of sorted specific T cells for metastatic melanoma patients

**DOI:** 10.1007/s00262-021-02961-0

**Published:** 2021-06-12

**Authors:** Brigitte Dréno, Amir Khammari, Agnès Fortun, Virginie Vignard, Soraya Saiagh, Tiffany Beauvais, Nicolas Jouand, Sylvain Bercegay, Sylvain Simon, François Lang, Nathalie Labarrière

**Affiliations:** 1 Dermato-Cancerology Department, CIC 1413, CHU Nantes, Nantes, France; 2 UTCG, CHU Nantes, Nantes, France; 3 CRCINA, Inserm, Université de Nantes, 44000 Nantes, France; 4 LabEx IGO “Immunotherapy, Graft, Oncology”, Nantes, France; 5 CHU Nantes, Nantes, France; 6 SFR Santé, CNRS, Inserm, Inserm UMS 016, CNRS UMS 3556, Université de Nantes, CHU Nantes, 44000 Nantes, France

**Keywords:** Adoptive cell transfer, Melanoma, TCR, Melan-A, MELOE-1

## Abstract

Adoptive cell transfer (ACT) of tumor-specific T lymphocytes represents a relevant therapeutic strategy to treat metastatic melanoma patients. Ideal T-cells should combine tumor specificity and reactivity with survival in vivo, while avoiding autoimmune side effects. Here we report results from a Phase I/II clinical trial (NCT02424916, performed between 2015 and 2018) in which 6 metastatic HLA-A2 melanoma patients received autologous antigen-specific T-cells produced from PBMC, after peptide stimulation in vitro, followed by sorting with HLA-peptide multimers and amplification. Each patient received a combination of Melan-A and MELOE-1 polyclonal specific T-cells, whose specificity and anti-tumor reactivity were checked prior to injection, with subcutaneous IL-2. Transferred T-cells were also characterized in terms of functional avidity, diversity and phenotype and their blood persistence was evaluated. An increase in specific T-cells was detected in the blood of all patients at day 1 and progressively disappeared from day 7 onwards. No serious adverse events occurred after this ACT. Clinically, five patients progressed and one patient experienced a partial response following therapy. Melan-A and MELOE-1 specific T-cells infused to this patient were diverse, of high avidity, with a high proportion of T lymphocytes co-expressing PD-1 and TIGIT but few other exhaustion markers. In conclusion, we demonstrated the feasibility and safety of ACT with multimer-sorted Melan-A and MELOE-1 specific T cells to metastatic melanoma patients. The clinical efficacy of such therapeutic strategy could be further enhanced by the selection of highly reactive T-cells, based on PD-1 and TIGIT co-expression, and a combination with ICI, such as anti-PD-1.

**Supplementary Information:**

The online version contains supplementary material available at 10.1007/s00262-021-02961-0.

## Introduction

Although having provided unprecedented benefit in the treatment of solid tumors, the use of anti-PD-1 antibodies, alone or in combination with other ICIs, does not exceed a therapeutic efficacy of 40% (monotherapy) to 60% (in combination). One possible reason for the therapeutic failure of ICIs in a significant proportion of patients may be the lack of a tumor-specific T-repertoire that can be mobilized by anti-IC antibodies. In this context, adoptive transfer of tumor-specific T cells could be a realistic option, especially in combination with ICI. For these strategies, ideal effector T-cells should combine different properties, such as tumor specificity and reactivity together with survival and tumor infiltration after transfer to autologous patients. In this context, we previously documented clinical responses in half of metastatic melanoma patients after adoptive transfer of T-cell clones specific for the Melan-A/MART-1 antigen [1, 2]. The lack of therapeutic efficacy in the other half of patients may have resulted from the injection of poorly reactive T cell clones in vivo due to the exhaustion status of infused T cells. Indeed, during the production process, the successive culture steps and the high number of cell divisions, required to get sufficient number of therapeutic T cell clones may cause exhaustion of some of these T cell clones. In addition, the infusion of T-cells specific for one single tumor antigen could promote tumor escape by favoring the growth of Melan-A negative tumor cells. These considerations led us to develop in 2015 a new ACT trial that combines tumor-reactivity of infused T-cells, polyclonality, and a shorter production process to improve T-cells survival in vivo. Moreover, we targeted two antigens to minimize tumor escape mechanisms by targeting a single specificity. At that time, anti-PD-1 had not yet become the standard of care for metastatic melanoma, so we did not immediately consider a combination therapy, this study being primarily a feasibility and safety study.

The two targeted antigens, Melan-A and MELOE-1, shared common features regarding their frequent expression in melanoma tumors, the recognition of two immunodominant HLA-A2 epitopes by melanoma-specific CD8 T cells involved in melanoma immuno-surveillance [3, 4], and of vast and diverse specific TCR repertoires in all HLA-A2 melanoma patients [5–7]. Indeed, blood frequencies of Melan-A and MELOE-1 specific T cells are, respectively, around 10^–4^ and 10^–5^ among CD8 T cells, which makes it possible to sort them from peripheral blood following a single step of in vitro peptide stimulation. These exceptionally high frequencies among T-cell repertoires specific for tumor antigens can be partly explained by a very strong bias in V-alpha usage in TCRs specific for these two epitopes [8, 9]. In addition, these two T cell repertoires also contain high avidity T cells making them relevant for a use in adoptive transfer. In terms of safety, Melan-A expression being restricted to the melanocytic lineage, the injection of T-cells specific reactive against this antigen may cause vitiligo by killing normal melanocytes which is an acceptable adverse effect considering the severity of metastatic melanoma [2]. Furthermore, results from previous ACT trials targeting Melan-A support the absence of other serious side effects [10, 11]. MELOE-1 antigen has never been targeted in any immunotherapy trial so far, but the very peculiar expression of this antigen led us to believe that the injection of MELOE-1-specific T-cells would be safe. Indeed, MELOE-1 antigen derives from one of the short ORFs of a lncRNA overexpressed in the melanocytic lineage [12, 13]. The IRES-dependent translation of MELOE-1 from this lncRNA is only effective in melanomas but not in melanocytes, conferring to this antigen a strict melanoma expression profile [14, 15]. Thus, the choice of these two antigen-specific T cell repertoires for melanoma immunotherapy appeared a realistic and relevant option. Based on these arguments and thanks to an original technique for sorting these specific T lymphocytes [16], we set up a unique phase I/II clinical trial called MELSORT (NCT02424916, https://clinicaltrials.gov), in which Melan-A and MELOE-1 specific T cells were infused to 6 metastatic melanoma patients. Here we report the characterization of infused T-cells, the characteristics of included patients and their clinical outcome, and the immune follow-up of infused T-cells.

## Material and methods

### Melanoma patients

**Fig. 1:**
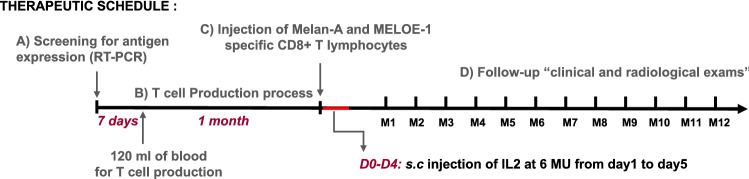
Therapeutic schedule of MELSORT clinical trial. **a** Antigen expression was validated by qPCR on a tumor biopsy from HLA-A*0201 metastatic melanoma patients, 7 days before the initiation of T cell production process. **b** Melan-A and MELOE-1 specific T-cells were produced in the Unit of cell Therapy of Nantes University hospital, from a sample of 120 mL of patient’s blood. **c** One month after the initiation of the production process, specific T cells were infused intravenously to the autologous patients, with subcutaneous injections of IL-2 (6 MU) during 5 days. **d** Patients were monthly followed clinically and radiologically

Treated patients were HLA-A2 melanoma patients, with unresectable stage III or IV melanoma, with tumors expressing Melan-A and MELOE antigens detected by RT-PCR. This prospective, open, monocentric phase I/II trial, promoted by Nantes University Hospital (France), was designed and conducted in accordance with the Declaration of Helsinki. The clinical protocol was approved by the ethics committee, and the national Health Agency (ANSM), and it was registered with the regulatory state authority (NCT02424916). All patients gave written informed consent before enrollment in the study and met the following inclusion criteria: male or female ≥ 18 and ≤ 75 years, patient expressing the HLA-A*0201 subtype of the human leukocyte antigen (HLA -A2), patient with metastatic melanoma stage IIIc or IV (AJCC 2010) except brain metastases, tumor expressing the antigens Melan-A and MELOE-1 detected by RT-qPCR, ECOG ≤ 1, lymphocyte count ≥ 1500/µL. Prior adjuvant melanoma treatment (before metastatic stage) were authorized (anti- BRAF, anti-CTLA4, IFN, TIL…). The primary objective was the evaluation of clinical and biological safety and the secondary objectives were the clinical efficiency and immune monitoring of infused T cells. The study design is illustrated by Fig. 1. After signed consent, patients were selected and 120 mL of blood were taken from HLA-A*0201 patients with tumor expressing Melan-A and MELOE antigens. One month after patients received intravenously autologous specific-T cells, with subcutaneous injections of IL-2 (6 MU, Chiron) from day 0 to day 4. From month 1 to month 12, patients were clinically (each month) and radiologically (every three months) followed-up and did not receive any other anti-cancer treatment (Fig. 1). The primary objective of safety was assessed by the NCI common toxicity criteria (Version 4.0, May 2009, http://ctep.cancer.gov). Overall tumor response (complete response, partial response, stable disease) was evaluated according to Response Evaluation Criteria in Solid Tumor (RECIST) and immune-related Response Criteria (irRC), every 3 months until M12.

Persistence of injected specific T cells evaluated by immune monitoring with HLA-peptide tetramers, at day 1, day 7, Day 30 and Day 90.

### Production of Melan-A and MELOE-1 specific T-cells from patient PBMC

Peripheral blood mononuclear cells (PBMC) were isolated by Ficoll-Hypaque gradient centrifugation, washed three times and seeded in 96 well/plates at 2 × 10^5^ cells/well in RPMI 1640 medium supplemented with 8% human serum (HS) (a pool from 20 donors prepared and secured by the EFS of Nantes) with 50U/mL of IL-2. PBMC were immediately stimulated with clinical grade peptides (CSBio, USA), either 1 µg/mL of Melan-A_A27L_ peptide (ELAGIGILTV) or 10 µg/mL of MELOE-1_36–44_ peptide (TLNDECWPA) for 14 days. Following stimulation, each microculture was evaluated for the percentage of specific CD8 T lymphocytes by double staining with the relevant APC-conjugated HLA-peptide tetramer (from the SFR Sante recombinant protein facility) and PE-conjugated anti-CD8 mAb (BD Biosciences, France) using a FACSCanto. Microcultures that contained at least 0.5% of Melan-A_A27L_ or MELOE-1_36–44_ specific T cells were selected, pooled and sorted with the relevant clinimers, as previously described [16]. These Clinimers were produced at GMP grade by the company Px-Therapeutics. Briefly, clinical grade M450-epoxy magnetic beads (Clin Ex-vivo Dynabeads, Life technologies) were covalently coupled to a monoclonal antibody specific for the peptide AviTag™ (Avidity, Aurora, CO, USA) that is fused to the heavy chain of the HLA constructs. Clinical batches of this Ab were produced by PX’Therapeutics from a master cell bank of clinical grade CHO-DG44 cell line (Life Technologies). HLA-A0201/peptide 3-mutated monomers were produced in GMP conditions by PX’Therapeutics. Final assembly of the Clinimer™ reagent was performed right before T cell sorting in the Unit of cell therapy, by incubating Chim-AvT beads with the appropriate HLA/peptide monomers (1 g of monomer for 10^7^ beads). The quality control of the resulting clinical grade multimers of HLA/peptide complexes (Clinimers™) was performed by staining with a PE-conjugated anti-HLA-A2 mAb (BD Biosciences, France).

**Fig. 2:**
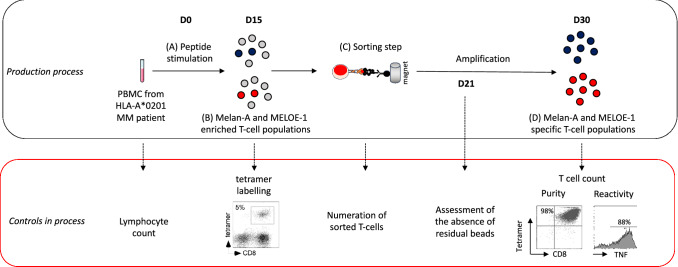
Methodology of melanoma-specific CD8 T cells production from HLA-A2 melanoma patients PBMC and controls in process. **a** PBMC from HLA-A2 melanoma patients were stimulated with each of the two antigen-derived peptide (Melan-A_A27L_ and MELOE-1_36–44_). **b** After 2 weeks, the amplification of specific-T cells is assessed by tetramer labelling. **c** Specific T-cells are sorted with HLA-peptide multimer-coated magentic beads, and amplified on irradiated feeders cells. **d** After 2 weeks, purity and reactivity of amplified T-cells on peptide-pulsed T2 cells are assessed and T-cells are infused to the patients

Sorted-specific specific T cells were seeded in 96 well plates for polyclonal amplification as previously described [17, 18] with irradiated feeder cells (PBMC pool from 4 donors and lymphoblastoid B cells-LAZ), clinical grade IL-2 (150 IU/mL) (Novartis Pharma, Rueil Malmaison, France), and PHA-L (1 µg/mL) (Sigma, Lyon, France), in 150 µL of RPMI1640 medium; containing 8% of HS. After a 14-days culture period, the total number of amplified T-cells is estimated by manual counting, and their specificity is assessed by double staining with the relevant APC-conjugated HLA-peptide tetramer (from the SFR Sante recombinant protein facility) and PE-conjugated anti-CD8 mAb (BD Biosciences, France). The purity threshold for cell injection has been set at 90% of cells labelled with the specific tetramer. Concerning the absolute number of infused T-cells, specifications had been fixed between 10^8^ and 5 × 10^8^ of each specificity, with an equal quantity of each T-cell population. Reactivity of amplified T cells was finally evaluated by TNF-α production in response to their cognate epitope. In brief, T cells were stimulated for 5 h in the presence of brefeldin A (10 µg/mL) with the appropriate peptide (10 µM for MELOE-1_36–44_ and 1 µM for Melan-A_A27L_). T-cells were then stained with APC-conjugated tetramer, fixed with 4% paraformaldehyde (Euromedex), permeabilized with PBS 0.1% saponin, intracellularly labelled with PE-conjugated anti-TNF-α mAb (BD Biosciences) for 30 min at room temperature and analyzed by flow cytometry. The reactivity threshold for cell injection has been set at 50% of cells producing TNF-α among tetramer-positive T-cells, and the cell viability threshold at 90%. The safety of the infused T-cells was controlled by evaluation of the residual magnetic beads by flow cytometry [16] in the cell suspension, the non-proliferation of the irradiated feed cells, the absence of mycoplasma and viruses as HHV6, HHV8, CMV and the absence of bacterial and fungal contamination. This production process and associated controls are summarized in Fig. 2. With the exception of patient P14 (who received only 6 × 10^7^ MELOE-1 specific T-cells), Melan A and MELOE-1 specific T-cells were finally collected in equal amounts (between 100 and 500 × 10^6^ cells for each specificity) up to 10^9^ total T-cells, and conditioned in a volume of 200 ml of 40 mg/ml of human serum albumin (Vialebex 40 mg/ml, LFB, France).

### TRBV repertoire diversity

Vß diversity of sorted Melan-A and MELOE-1 specific T cell lines was analyzed by labeling with 24 anti-Vß mAbs included in the IOTest Beta Mark TCR V Kit (Beckman-Coulter, IM3497). These cytometric analyses were performed on a Facs Canto II (BD Biosciences). We followed throughout the manuscript the nomenclature of IMGT database [19].

For TCR sequencing of MELOE-1-specific T cells from P5 patient, total RNA was extracted from 5 × 10^5^ specific-T cells using QIAGEN RNeasy Kit. 25 ng of RNA was used to build libraries with the QIAseq Immune Repertoire T-cell Receptor Panel (Catalog 333705—IMHS-001Z), as previously described [9]. FASTQ files were analyzed in the QIAGEN GeneGlobe Data Analysis Center using the Immune Repertoire Application. The clonotype calls are generated using the IMSEQ software [20]. Clonotypes were defined on the basis of unique amino-acid sequences of CDR3 beta regions. In our set of data, the total number of unique TCR sequences was identical to the number of clonotypes.

### Cell lines

The human TAP deficient cell line T2 (174 × CEM.T2) used as a presenting cell was purchased from the ATCC (CRL-1992). Melanoma cell lines, registered in the Biocollection PC-U892-NL (CHU Nantes, France), were established from metastatic tumor fragments in the Unit of Cell therapy of Nantes. All these cells were cultured in RPMI1640 medium supplemented with 10% of Fetal Bovine Serum (Eurobio), 2 mM L-glutamine (Gibco), 100 U/mL penicillin (Gibco) and 0.1 mg/mL streptomycin (Gibco).

### Functional avidity and melanoma reactivity

Functional avidity of antigen-specific sorted T-lymphocytes was evaluated after co-culture with TAP-deficient T2 cells loaded with a range of either Melan-A_A27L_ (ELAGIGILTV) or MELOE-1_36–44_ peptides at the effector/target ratio 1/2, through the measurement of CD107a mobilization. Reactivity of sorted T-cell populations was also evaluated on 3 HLA-A*0201 melanoma cell lines, at various effector/target ratios, through the measurement of CD107a mobilization. CD107a mobilization was measured after a 3 h. of co-culture at 37 °C in the presence of a CD107a-specific mAb (H4A3 clone, BioLegend). T lymphocytes were then stained with anti-CD8 antibodies (Clone RPA-T8, BioLegend) and analyzed by flow cytometry on a Facs Canto II (BD Biosciences).

### RT-qPCR for *MLANA* and meloe expression in melanoma cell lines

Total RNA was extracted from tumors or melanoma cell lines by RNA purification system NucleoSpin RNA II (Macherey–Nagel, Hoerdt-FRANCE) (RNA integrity number > 7). Retrotranscription was performed using 1 μg of total RNA, oligodT, and SuperScript III reverse transcriptase (Invitrogen-Life-Technologies, Saint-Aubin-FRANCE). Relative quantification of *meloe*, *MLANA*, and housekeeping genes (HKG) RPLPO and cyclophilin-A expression was performed using Brilliant SYBR Green qPCR (Stratagene-Agilent Technologies, Les-Ulis-FRANCE). cDNA samples (20 ng) were added to SYBR Green Master Mix with specific primers at 200 nM. RPLPO: 5′-GTGATGTGCAGCTGATCAAGACT-3′ and 5′-GATGACCAGCCCAAAGGAGA-3′; cyclophilin-A: 5′-CCACCGTGTTCTTCGACAT-3′ and 5′-CCAGTGCTCAGAGCACGAAA-3′; meloe: 5′-GTCCTCCCCAGCACCAGAGT-3′ and 5′-AGCCTGCCATCTGCAATCCT-3′; MLANA: 5′-TGCTCATCGGCTGTTGGTATTG-3′ and 5′-GGAGCATTGGGAACCACAGG-3′. For the four genes, thermal cycling was 95 °C for 10′, 40 cycles at 95 °C for 30″, 60 °C for 1′, and 72 °C for 1′. The efficiency of PCR reaction was validated with duplicate series of tenfold-diluted cDNA from the melanoma cell line M113, performed in parallel to plot the standard curves for the three genes. Mean threshold cycle (CT) values from duplicate PCR reactions were normalized to mean CT values for the two HKG from the same cDNA preparations. The relative expression ratio of a target gene was calculated based on the PCR efficiency (*E*) and the CT deviation between a given tumour or melanoma cell line (*x*) and a calibrator (M113 melanoma cell line), expressed in comparison with the mean of the HKG: ratio = (*E* target) ^ΔCT target (calibrator − *x*)^/mean ((*E* HKG) ^ΔCT HKG (calibrator − *x*)^).

### Phenotypic characterization of infused T cells

Therapeutic cells were thawed in RPMI (Gibco) containing 8% human serum, washed twice, separated in two tubes and stained in 30 µL of staining buffer (PBS, 0.1% BSA) containing 7AAD (BD Biosciences), a specific tetramer (HLA-A2/Melan-A or HLA-A2/MELOE-1 conjugated to PE) and antibodies from panel 1 and 2 and incubated for 1 h at 4 °C. Cells were then washed twice before acquisition on a BD Symphony A5.2 analyzer. Data were collected and analyzed using the BD FACS Diva Software v8.05.

- *Panel 1 (activation/exhaustion)* TIGIT (PE-Cy7), PD-1 (BV421), KLRG1 (APC), CD38 (BV480), CD39 (BUV737), CD62L (FITC), CD103 (BB700).
- *Panel 2 (Chemokine receptors)* CCR6 (PE-Cy7), CXCR3 (BV421), CCR9 (PE), CXCR4 (BV711), CXCR5 (BV605), CCR10 (BB515), CD103 (BV480).

### Immune follow-up of infused T cells

To minimize non-specific staining, HLA-A2-Melan-A and MELOE-1 tetramers were tittered and used at the lowest concentration that showed a clearly distinguishable positive T-cell population [1]. Total blood (3 mL) from melanoma patients at different time-points were incubated with 10 µg/mL of either Melan-A or MELOE-1/HLA-A*0201 APC-tetramers and with PE-conjugated anti-CD8 (Biologend) and FITC-conjugated anti-CD3 (Biolegend), for 1 h at 4 °C in the dark, with gentle agitation every 15 min. After incubation, the red blood cells are lysed through incubation in 10 volumes of lysis solution (BD Biosciences), during 15 min at room temperature. After washing in PBS-0.1%BSA, cells were resuspended in PBS and analyzed on a FACSCanto. Frequencies of antigen-specific T-cells are calculated based on the number of tetramer positive cells, divided by the total number of CD3^+^/CD8^+^ T lymphocytes analyzed.

## Results

### Patients pre-included and treated by ACT with melanoma-specific sorted T cells

Twenty metastatic melanoma patients were pre-selected for this clinical trial (Table S1), and finally 6 patients were treated by ACT with T-cells specific for the two selected melanoma antigens (Table 1). Indeed, 7 patients did not meet the inclusion criteria, such as improper HLA-A2 allele, absence of antigen expression by the tumor, or severe lymphopenia which hampered the production of sufficient numbers of specific T-lymphocytes.

**Table 1:** Clinical characteristics and outcome of the 6 HLA-A*0201 metastatic melanoma who received ACT in the MELSORT trial

Patients	Age Sex	Tumor type^a^	BRAF status	AJCC Stage	ECOG	Breslow Ulceration	LDH IU/L	Melan-A and MELOE-1 relative expression^b^	Date of ACT	Previous treatment	RECIST evaluation (M12)
P5 PM	71 W	ALM	WT	IIIC	0	11 mm No	193.2 (1 × N)	Melan-A: 0.35 ± 0.11 MELOE-1: 1.70 ± 0.5	08-Dec 2015	None	PR
P10 RC	58 M	NM	WT	IIIC	0	10 mm Yes	167.4 (1 × N)	Melan-A: 0.63 ± 0.26 MELOE-1: 1.14 ± 0.47	25-Oct-2016	IFN (Apr-May 2015) Nivolumab (Nov-2015 to Sept 2016)	PD (M6)
P13 TV	68 W	Unknown	WT	IVM1b	1	Unknown	183 (1 × N)	Melan-A: 1.78 ± 0.83 MELOE-1: 2.61 ± 0.26	15-Nov-2017	None	PD (M3)^c^
P14 CO	79 W	NM	V600E	IIIC	0	3.8 mm Yes	195 (1 × N)	Melan-A: 0.41 ± 0.05 MELOE-1: 0.25 ± 0,03	27-Feb-2018	None	PD (M3)
P16 CM	74 W	SSM	WT	IIIC	0	6 mm Yes	152.4 (1 × N)	Melan-A: 0.14 ± 0.02 MELOE-1: 0.97 ± 0.15	24-Apr-2018	None	PD (M6)
P17 MF	43 M	NM	WT	IIIC	1	1.7 mm Unknown	249 (1.1 × N)	Melan-A: 0.004 ± 0.002 MELOE-1: 0.44 ± 0.5	15-May-2018	IFN (Jan 2011- Jul 2012) IPI + Nivo (Nov 2017- Feb 2018)	PD (M3)

^a^*SSM* Superficial spreading melanoma, *ALM* acrolentiginous melanoma, *NM*: Nodular melanoma

^b^Melan-A and MELOE-1 relative expression was calculated based on the PCR efficiency and the Ct deviation between a given tumor and a reference melanoma cell line (M113), expressed in comparison with the expression of two HKG.

^c^This patient exhibited a complete response after 9 cures of flat doses of Nivolumab (flat dose) from March 2018

In five patients, the production process was unsuccessful either because of a too low frequency of antigen-specific circulating T cells (for 3 patients), a mishap in the sorting procedure for Melan-A-specific T cells (one patient), or an insufficient amount and reactivity of MELOE-1-specific T-cells (one patient). Of note, one of the patients exhibiting a too low frequency of MELOE-1-specific T-cells (P4) received 5 × 10^8^ Melan-A-specific CD8 T-cells as a compassionate treatment. Patient P11 could not receive the treatment due to a general status alteration during the production process that required transfer to palliative care and patient P15 could not benefit from the treatment because the regulatory agency was considering an amendment to the protocol, and did not give its authorization within the deadline.

All but one of the 6 patients who received the complete treatment were stage IIIC metastatic patients (Table 1), with tumors expressing the two targeted antigens. Four of these patients received antigen-specific T lymphocytes as a first line of treatment, which was conceivable at that time, considering that recommendations for the use of anti-PD-1 as first-line therapy did not come until May 2016, and considering the progressive capacity of melanoma in these patients. The two other patients had been treated previously either with Nivolumab or a combination of Nivolumab and Ipilimumab.

### Production of therapeutic T cells and controls

Melan-A and MELOE-1-specific T-cells were produced from HLA-A*0201 PBMC from melanoma patients, according to a validated procedure [16]. After a peptide stimulation step of PBMC, the frequency of individual microcultures containing specific-T cells was assessed through CD8/tetramer double labelling (Fig. 2 and Table 2). Positive microcultures were grouped and specific-T cells were sorted with magnetic beads coated with each relevant tetramer, called Clinimers™ (Fig. 2).

**Table 2:** Characteristics of infused antigen-specific CD8 T lymphocytes

Patients	Positive microcultures		Number of sorted-T cells^a^		Number of amplified T-cells^b^		Amplification factors^c^		Purity^d^		Reactivity^e^		ACT treatment^f^	
	Melan-A	MELOE-1	Melan-A	MELOE-1	Melan-A	MELOE-1	Melan-A	MELOE-1	Melan-A (%)	MELOE-1 (%)	Melan-A (%)	MELOE-1 (%)	Melan-A	MELOE-1
P5 SM	16/192	21/384	9.7 × 10^4^	2 × 10^5^	8.38 × 10^9^	1.15 × 10^9^	86 392 *(16 div.)*	5 750 *(12 div.)*	99.6	98.3	92.6	86.5	5 × 10^8^	5 × 10^8^
P10 RC	26/96	31/672	5.5 × 10^4^	9.6 × 10^4^	2.41 × 10^9^	3.02 × 10^9^	43 818 *(15 div)*	31 458 *(15 div)*	98.3	94.1	78.7	61.9	5 × 10^8^	5 × 10^8^
P13 TV	98/384	10/576	1.2 × 10^5^	8.8 × 10^4^	7.24 × 10^8^	3.27 × 10^9^	6 033 *(12 div)*	37 159 (*15 div)*	99.4	99.8	87.6	86.4	5 × 10^8^	5 × 10^8^
P14 CO	48/288	12/480	1.44 × 10^5^	2.02 × 10^5^	3.6 × 10^8^	6.6 × 10^7^	2 083 *(11 div.)*	327 *(8 div.)*	99.3	97.4	88.3	80%	3 × 10^8^	6 × 10^7^
P16 CM	127/288	76/384	5 × 10^4^	2.5 × 10^4^	4.7 × 10^8^	3.6 × 10^8^	9 400 *(13 div.)*	14 400 *(14 div.)*	94.9	97.8	83.5	80.1	3.6 × 10^8^	3.6 × 10^8^
P17 MF	202/288	34/384	7.2 × 10^4^	2.04 × 10^5^	3.3 × 10^8^	1.32 × 10^9^	4 583 *(12 div.)*	6 470 *(12 div.)*	99.4	99.3	83.2	66.2	3.1 × 10^8^	3.1 × 10^8^

^a^Numbers of specific-T cells (rosetted) amplified on feeders cells, after the sorting step

^b^Total numbers of specific-T cells obtained after the amplification step

^c^Amplification factors calculated from total numbers of amplified T cells divided by the numbers of sorted T-cells. The approximate number of cell divisions is indicated between brackets

^d^Purity of amplified specific T-cells was assessed through double-labelling with anti-CD8 mAb and each specific HLA-peptide tetramer. The compliance of this specification has been set above a threshold of 90%

^e^Peptide-reactivity of amplified T-cells was measured by TNF-α production after a 5 h stimulation period with each specific peptide. The compliance of this specification has been set above a threshold of 50% of TNF-producing T-cells among tetramer-positive cells

^f^Numbers of specific-T cells infused to each patient

The number of sorted-T cells was estimated (Table 2) and specific T lymphocytes were further amplified on feeder cells. At day 24 of the process (± 2 days), the absence of residual magnetic beads in the cell cultures was assessed by flow cytometry [16]. Finally, at the end of the amplification process (D30), amplified T cells were enumerated. As shown in Table 2, T lymphocytes underwent between 8 and 16 divisions during this amplification step after stimulation with the cognate peptides. Cell viability was always above 90%, and the purity of the amplified T cells always above 94% (assessed by tetramer labelling). The reactivity of specific T-cells was always above 60% as assessed by the production of TNF-α upon peptide stimulation.

The research for mycoplasma, viral (HHV6, HHV8 and CMV), bacterial and fungal contamination was always negative, as well as the presence of residual magnetic beads in cell suspension.

All 6 patients but one received equal numbers of Melan-A and MELOE-1 specific T lymphocytes (between 3.1 × 10^8^ and 5 × 10^8^ of each specificity). Patient 14 received 3.6 × 10^8^ Melan-A-specific T cells but only 6 × 10^7^ MELOE-1-specific T cells due to a low number of divisions of these T cells during the amplification process (only 8 divisions within 15 days of amplification) (Table 2). This patient was therefore the only patient to have received less than 10^8^ T cells of this specificity. The decision to treat him despite this non-compliance was justified by the rapid progression of his disease and by the availability of Melan-A specific cells in sufficient numbers. Not to treat this patient would have been a loss of opportunity, given the favorable benefit-risk ratio.

### Side effects and clinical outcome

Regarding the safety, no serious adverse events related to the treatment were reported during this study. The only observed adverse events were mild to moderate and are summarized in the Figure S1, classified according the system organ class (SOC). Overall, the most represented SOC among the reported events were general manifestations, consistent with the manifestations observed during IL-2 injection, such as asthenia, flu-like syndrome and pain at site injection, followed by gastrointestinal disorders. All the reported adverse events related to IL-2 treatment had resolved by the month following the last cytokine injection, as previously observed with TIL and IL-2 injection [21].

Regarding the clinical efficacy, among the 6 patients who received the treatment, one experienced a partial response according to RECIST criteria (Table 1). The other patients progressed either at month 3 or month 6. Of note, one of the progressing patient received anti-PD-1 treatment 4 months after ACT and experienced a complete response after 9 cures, still ongoing (P13).

### Frequency and diversity of therapeutic T lymphocytes

**Fig. 3:**
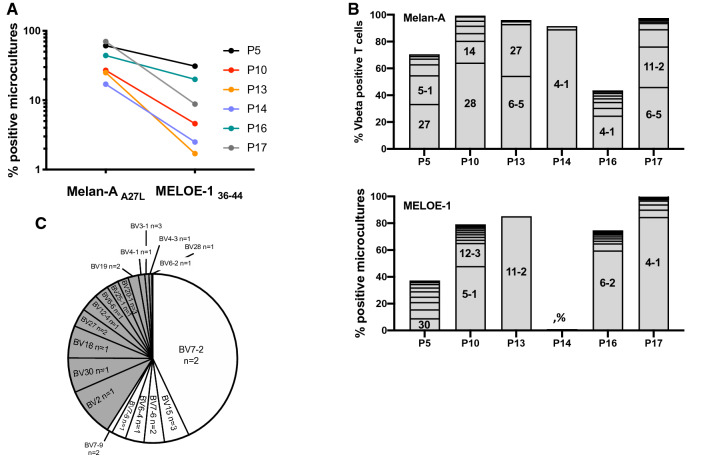
Frequency and Vbeta diversity of Melan-A and MELOE-1-sorted T cells. **a** Percentages of microcultures containing antigen-specific T cells after the step of peptide-stimulation. **b** TRBV diversity of sorted-antigen specific T cells, determined by flow cytometry. Each stack represents a distinct TRBV subfamily, and dominant TRBV chains are indicated within the corresponding stack. ND: Not determined. **c** Cumulative frequencies (UMI counts) of each TRBV chains in the MELOE-1 specific T cell repertoire from P5 patient. The number of clonotypes using a given TRBV chain is indicated

The frequency of microcultures containing antigen-specific T cells after in vitro peptide stimulation was calculated as the ratio between wells containing at least 1% of specific T-cells and the total number of stimulated microcultures. On this basis, the frequency of microcultures containing Melan-A-specific T cells ranged between 17 and 70%, whereas that of those containing MELOE-1 specific T cells ranged between 1.7 and 31% (Fig. 3a). For each individual patient, the frequency of MELOE-1 specific T cells was always lower than that of Melan-A-specific T cells, as previously observed in healthy donors and melanoma patients [5]. Patients P5 and P16 exhibited the highest frequencies of Melan-A and MELOE-1 specific T cells (Fig. 3a). The diversity of antigen-specific sorted T-cell populations based on the frequency of Vß subfamilies was further documented for each patient (Fig. 3b) with a panel of 24 specific Vß-specific antibodies, covering the most frequently expressed TRBV chains. As shown in Fig. 3b and Table 3, Melan-A and MELOE-1-sorted T-cell populations expressed between 2 and 8 TRBV chains and 1–10 TRBV chains, respectively, with a frequent dominant expression of one or two TRBV chains. We could not document complete antigen-specific TRBV repertoire for P5, P14 and P16 patients (less than 50% of TRBV repertoire identified), due to the lack of available antibodies against frequently expressed TRBV chains within Melan-A and MELOE-1 T cell repertoires (especially for TRBV6 and TRBV7 families) [9]. To partially answer this question, we analyzed the cumulated frequencies of TRBV chains within MELOE-1-specific T cells from P5 patient by TCR sequencing (Fig. 3c). This parameter illustrates the relative abundance of CDR3ß clonotypes using a given TRBV gene, within a given repertoire. In this TCR repertoire, we identified 31 CDR3ß clonotypes, and this analysis indeed confirmed that the most abundant clonotypes expressed TRBV chains for which the specific antibodies were not available in the antibody panel (white sections in Fig. 3c).

**Table 3:** Diversity, EC50 and melanoma reactivity of antigen-specific T-cells

	Vbeta diversity^a^		EC50 (M)^b^		Reactivity on M134 cell line^c^	
	Melan-A	MELOE-1	Melan-A_A27L_	MELOE-1_36–44_	Melan-A (%)	MELOE-1 (%)
P5 SM	6	9*	3.4 × 10^–13^	1.6 × 10^–11^	92	71
P10 RC	7	12	1.3 × 10^–11^	2.1 × 10^–10^	23	7
P13 TV	4	1	4.4 × 10^–14^	4.6 × 10^–10^	92	4
P14 CO	2	ND*	2.5 × 10^–12^	2.1 × 10^–11^	44	66
P16 CM	8*	10	7.7 × 10^–12^	5.3 × 10^–10^	21	28
P17 MF	8	8	2.3 × 10^–11^	1.0 × 10^–10^	24	78

*Indicates T-cell populations for which we were only able to identify less than 50% of the total TRBV repertoire due to missing specific antibodies

^a^Number of Vbeta subfamilies identified by a panel of 24 Vß-specific mAb

^b^EC50 were determined after activation of antigen-specific T cell lines by T2 cells loaded with a range of Melan-A_A27L_ or MELOE-1_36–44_ peptides

^c^reactivity against M134 cell line (strongly expressing Melan-A and MELOE-1 antigens) was assessed by measuring the % of CD107a degranulation, after a 3 h-stimulation period with melanoma cells, at ratio 1/1

### Functional avidity and reactivity of antigen-specific T lymphocytes

**Fig. 4:**
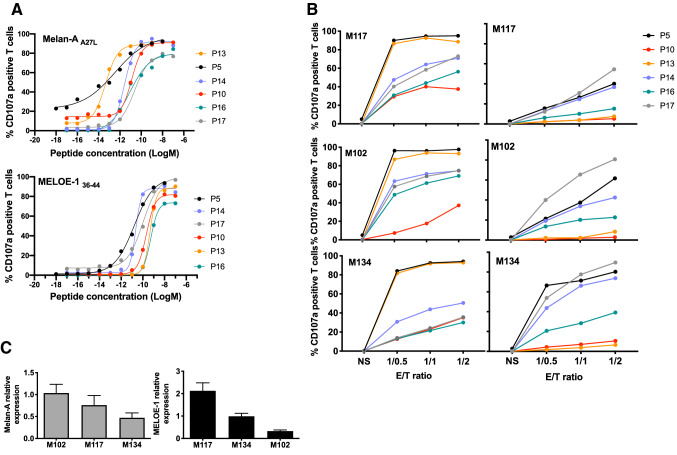
Functional avidities of Melan-A and MELOE-1-specific CTL lines and PD-1 expression. **a** CD107a expression by Melan-A -specific CTL lines, in response to TAP-deficient T2 cells loaded with a range of Melan-A_A27L_ (up) or MELOE-1_36–44_ (bottom) peptides. **b** Anti-tumor reactivity of Melan (left) and MELOE-1 (right)-specific CTL lines was validated through the measurement of CD107a degranulation in response to 3 HLA-A2 melanoma cell lines expressing the two antigens, at 3 different E/T ratios. **c** *mlana* (left) and *meloe* (right) genes relative expression were calculated from the Ct value normalized on the Ct value of of a reference melanoma cell line, and on the Ct value of two HKG (cyclophilin and RPLPO)

We tested the functional avidity of each antigen-specific T-cell population on T2 cells loaded with a range of each cognate peptide, using double staining with a specific anti-CD8 Ab together with CD107a labeling (Fig. 4a). As illustrated by Fig. 4a (upper panel) and Table 3, the range of functional avidities for Melan-A-specific T-lymphocytes was between 10^−11^ M and 10^−14^ M, whereas this range was lower and much narrower for MELOE-1 specific lymphocytes, ranging from 10^−10^ M and 10^−11^ M (Fig. 4a, lower panel and Table 3). Interestingly, with the exception of P13 patient, there was a relative proportionality between the EC50 of Melan-A and MELOE-1 specific lymphocytes for a given patient. As an example, Melan-A and MELOE-1 specific T lymphocytes from patient P5 both exhibited high functional avidities (black curves in Fig. 4a), whereas these T-lymphocytes from patient P16 both exhibit the lowest functional avidities (green curves in Fig. 4a). We also tested the functional avidity of the main TRBV subfamilies within a given T-cell repertoire (Figure S2). Globally, there were no drastic differences between the functional avidities of total CD8 T cells and that of dominant TRBV subfamilies. As expected, the reactivity of these T-cell populations, measured on HLA-A*0201 melanoma cell lines (Fig. 4b) were mostly consistent with their functional avidities. Melan-A-specific T-cells from P5 and P13 patients exhibited the highest reactivity against the 3 tested melanoma cell lines, compared to the 4 other populations (Fig. 4b, upper panel and Table 3). This difference was especially marked against M134 cell line, that displayed the lowest level of Melan-A expression, measured by RT-qPCR (Fig. 4c, upper panel). MELOE-1-specific T-lymphocytes from P5 patient were also among the most reactive against the three melanoma cell lines, with T-cells from P17 and P14 patients (Fig. 4b, lower panel). Conversely, MELOE-1 specific-T-cells from P10, P13 and P16 patients barely recognized these cell lines, consistent with their lower functional avidity against the MELOE-1 peptide (Fig. 4a, lower panel).

In conclusion, these results showed that therapeutic T-cell infused to P5 patient, who exhibited a partial response (Table 1) displayed the best profile, both in terms of functional avidity of Melan-A and MELOE-1 specific T cells and of reactivity against target melanoma cells expressing the two antigens.

### Activation/exhaustion markers expressed by therapeutic T-cells

We previously described that functional avidity of Melan-A-specific T cells was associated with the co-expression of PD-1 and TIGIT receptors [22]. This T cell subset thereafter called DPOS subset is an heterogeneous T cell population containing both highly activated T cells and exhausted T cells [23]. We therefore investigated through multiparametric flow cytometry the proportion of DPOS T cells in each antigen-specific T cell population, together with their expression of additional exhaustion markers, CD39, KLRG1 and CD103. The gating strategy is depicted in Figure S3, by a representative example.

**Fig. 5:**
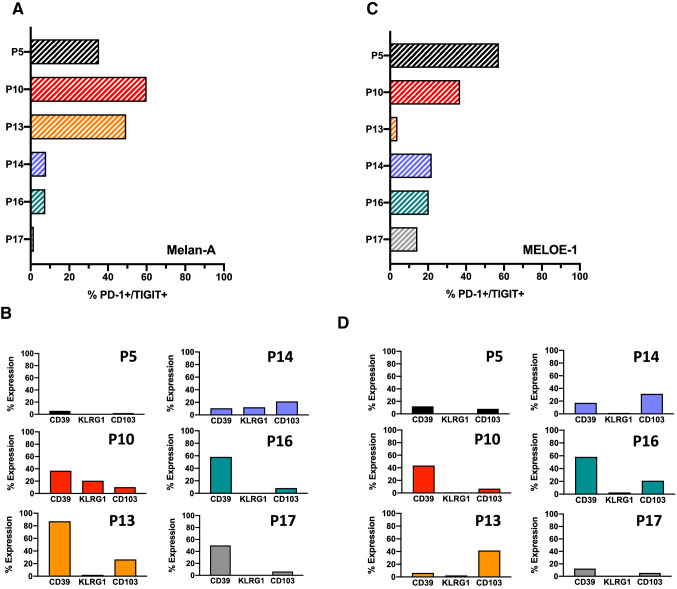
Fraction of PD-1^+^/TIGIT^+^ T cells among specific-T cells and exhaustion markers expressed by this subset, analyzed on a BD-Symphony cytometer. **a** and **c** Fraction of PD-1^+^/TIGIT^+^ (DPOS) T cells among Melan-A and MELOE-1 infused specific T-cells. **b** and **d** Percentages of Melan-A and MELOE-1 DPOS T cells expressing CD39, KLRG1 and CD103 markers

Three Melan-A specific T cell populations (from P5, P10 and P13) contained high proportion of DPOS T cells, above 30% (Fig. 5a) and among these 3 populations, those from P10 and P13 patients both expressed high levels of CD39, together with KLRG1 and CD103 for P10 patient and CD103 for P13 patient (Fig. 5b). Conversely, Melan-A-specific T cells from P5 patient almost did not express any of those molecules. In the three other Melan-A specific T cell population, the fraction of DPOS T cells is low (Fig. 5a), and these subpopulations all expressed CD39 with KLRG1 and CD103 for P14 patient (Fig. 5b).

For MELOE-1-specific T cells, 2 populations (P5 and P10) contained more than 30% of DPOS T cells (Fig. 5c). Among these two subpopulations, only those from P10 contained a high proportion of CD39 positive T lymphocytes (Fig. 5d). MELOE-1 specific T cells from P14 and P16 patients contained around 20% of DPOS T-cells, enriched with significant fractions of CD39^+^ and CD103^+^ T lymphocytes. Finally, MELOE-1-specific T-cells from P17 and especially P13 patients contained low fractions of DPOS T cells. These results, together with the functional avidities and the tumor reactivity of these therapeutic T cells suggested that the presence of a high proportion of DPOS T cells lacking expression of exhaustion markers, especially CD39, could condition therapeutic efficacy.

### Immune follow-up of infused antigen-specific-T cells in the blood of patients

**Fig. 6:**
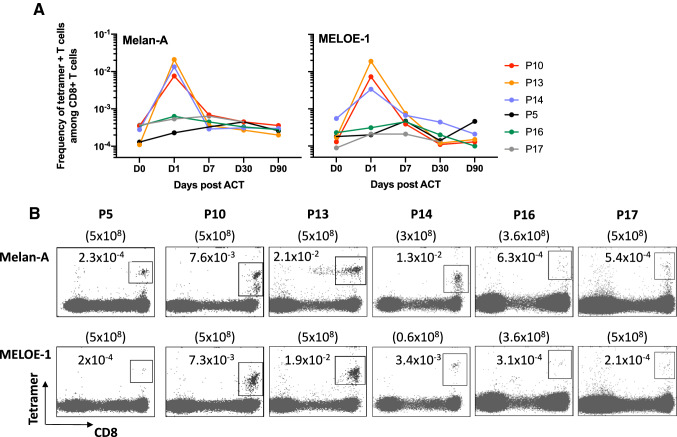
Immune follow-up of infused antigen-specific T lymphocytes. **a** Frequency of Melan-A and MELOE-1-specific T cells among CD8 T cells detected in patients’ blood across time points post ACT. **b** Dot plots illustrating the presence of tetramer-positive T-lymphocytes, at day 1 post-injection, through multiple labelling performed on 3 mL of total blood. Frequencies of specific-T cells are indicated in the dot plots and upper numbers within brackets indicate the amount of infused T cells.

Another feature associated with clinical efficacy of ACT is the in vivo persistence of infused T-cells and their ability to migrate to the tumors. In order to evaluate blood persistence of infused cells, we measured the frequency of circulating antigen-specific T cells from day 1 to day 90 after ACT, by labeling 3 ml of whole blood with HLA-peptide-specific tetramers and CD3 and CD8-specific antibodies. Frequencies of specific T-cells were calculated among total CD8 T cells. Before T cell transfer, the frequencies of tetramer positive cells, respectively, ranged from 1.3 × 10^–4^ to 3.7 × 10^–4^ for Melan-A-specific T-cells and between 8.9 × 10^–5^ and 5.5 × 10^–4^ for MELOE-1-specific T-cells. (Table S2 and Fig. 6a). After infusion, these frequencies increased significantly at day 1 in all treated patients (Table S2 and Fig. 5a), with the corresponding whole blood staining shown in Fig. 6b. However, two groups of patients could be clearly distinguished at D1: patients P10, P13 and P14 had very high frequencies of specific cells in the blood (up to 10^–2^) while in patients P5, P16 and P17 the majority of the infused cells already disappeared from the blood. We were thus prompted to look for possible differences in homing markers or chemokine receptors that may explain these two different profiles. We performed a multipanel labelling of the infused T cells to analyze the expression of CD62L, CXCR4, CCR10, CCR9 and CCR6 (Figure S4) but we could not identify any differential expression that could account for these different behaviors.

## Discussion

This study demonstrates the feasibility and safety of adoptive transfer of T lymphocytes specific for two melanoma antigens, selected via their TCR by an original method [16]. An advantage of this method is its relative simplicity of implementation, its speed and safety, with no need for genetic modification, as compared with the production of CAR T-cells or TCR transfer. In addition, selecting therapeutic cells from the endogenous repertoire naturally limits the range of their functional avidity, which may also reduce the severe autoimmune off-target toxicities sometimes observed with engineered T cells with very high affinities [24, 25]. Nevertheless, one of the prerequisites for successful production of antigen-specific T cells is the frequency of the corresponding T cell repertoires in the peripheral blood of patients. As such, Melan-A and MELOE-1 antigen-specific T repertoires are among the most frequent for tumor antigen-specific T repertoires [5, 9]. Indeed, blood frequencies of Melan-A and MELOE-1 specific T cells are, respectively, around 10^–4^ and 10^–5^ among CD8 T cells in HLA-A*0201 melanoma patients, allowing their sorting from patient blood after a single step of peptide stimulation. This production method may thus require some minor adjustments for the amplification of less frequent T cell repertoires, such as those specific for some cancer germline antigens or neo-antigens. In this respect, we previously demonstrated that the multimer sorting procedure remained efficient to sort much rarer repertoires, such as gp100 or NA17-A specific T cells [26].

Despite this relatively high peripheral frequency, for 2 of the 11 patients for whom the production process of specific T lymphocytes was initiated, stimulation by the MELOE-1_36–44_ peptide did not allow the amplification of specific lymphocytes, probably due to less frequent T-cell repertoires in these two patients (BM-02 and SE-04). Of note, in one of these two patients (BM-02), the peptide stimulation step also failed to amplify specific Melan-A T lymphocytes, which is more surprising in view of the high frequency of this T-cell repertoire in all HLA-A*0201 patients. Melan-A_A27L_ peptide stimulation also failed to amplify specific T-cells from another patient (SC-01), while the amplification of MELOE-1 specific T-cells was successful for this same patient. For these patients, we can hypothesize an exhaustion of Melan-A specific T-cells, due to chronic restimulation, as observed for virus-specific T lymphocytes in pathological contexts [27].

In addition to the amplitude of their specific T repertoires, Melan-A and MELOE-1 antigens were also selected on the basis of their frequent and shared expression by melanoma tumors, and regarding MELOE-1 antigen, for its strict tumor specificity. Indeed, this antigen, unlike Melan-A, had never been targeted in melanoma immunotherapy trials, and it was crucial to guarantee the safety of injecting T lymphocytes specific for this antigen. This non-toxicity was, if not totally guaranteed, at least strongly supported by the dual expression control of this antigen, both at the transcriptional level, which limits its expression in the melanocyte lineage [12], and at the translational level, with a protein expression controlled by an IRES sequence, active only in melanomas [14, 15]. No side effects were observed for the 6 treated patients, apart from the expected side effects related to IL-2 injections, carried out to promote the survival of the injected T lymphocytes [10]. For all treated patients, we observed increased frequencies of tetramer-specific T cells in blood the day after T-cell infusion, with marked increase for three of them (Fig. 6). These T cells progressively disappeared from blood at day 7 that may reflect their migration to secondary lymphoid organs and tumors, as previously shown after the injection of Melan-A specific T-cells [11].

Another major point for the effectiveness of adoptive transfer is the functional avidity of the injected T-lymphocytes. As the MELOE-1 antigen is not expressed in the thymus, T lymphocytes specific for this antigen are not subject to thymic selection, thus favoring the presence of T lymphocytes with high functional avidity within this T cell repertoire. The same is true for the Melan-A specific T repertoire, despite the fact that Melan-A has long been considered as a self-antigen tolerized in the thymus. Indeed, although Melan-A mRNA can be detected in medullary thymic epithelial cells, it was documented that in the majority of individuals, this mRNA is truncated due to a mis-initiation of gene transcription [28]. In the truncated mRNA transcripts, the immunodominant HLA-A2 Melan-A_26-35_ epitope is lost thus leading to the evasion of central self- tolerance towards this epitope. Functional avidities of Melan-A and MELOE-1 sorted-T cells were assessed using T2 cells pulsed with the Melan-A analog peptide (A27L) or the MELOE-1_36–44_ peptide. Among Melan-A specific T-cells, T lymphocytes from P5 and P13 patients exhibited the highest functional avidity, followed by T cells from P10 and P14, and from P16 and P17 patients (Fig. 4a and Table 3). The range of functional avidities is much narrower for MELOE-1 specific T cells, with T cells from P5, P14 and P17 exhibiting better EC50 on the natural peptide than T cells from P10, P13 and P16 patients (Fig. 4a and Table 3). Based on these results, it is interesting to observe that only lymphocytes from the single responding patient (P5, black curves in Fig. 4a) displayed high avidities against both Melan-A and MELOE-1 peptides. These results were further confirmed by measuring the reactivity of T cells against HLA-A2 melanoma cell lines, spontaneously expressing the two target antigens (Fig. 4b and c). Again, Melan-A specific T cells from P5 and P13 patients were the most reactive against melanoma cell lines, as well as MELOE-1-specific T cells from P5, P14 and P17 (Fig. 4b). Besides patient P5, lymphocytes of both specificities from patient P14 also exhibited good functional avidity and were reactive against melanoma cells (blue curves and histograms in Figs. 4a and 4b). Nonetheless, P14 patient did not benefit from ACT and the major difference when compared to patient P5 was the poor diversity of its T cell repertoire (Fig. 3b). Indeed, Melan-A-specific T cell repertoire from P14 patient, was dominated by a single Vbeta subfamily (probably mainly composed of a dominant clonotype), and we could not identify the Vbeta subfamilies that constitute MELOE-1-specific T-cell repertoire, also suggesting the dominance of a clonotype not recognized by the available Vbeta-specific antibodies. In contrast, Melan-A and MELOE-1 specific T-cell repertoires from P5 patient were rather diverse, with, respectively, 6 and more than 9 Vbeta subfamilies (Fig. 3b and Table 3). More specifically, through TCR sequencing, we could identify 31 distinct clonotypes within MELOE-1 specific T-cells from this patient (Fig. 3c). The diversity of a given T-cell repertoire increases the probability of success of adoptive cell transfer, in particular by diversifying the expression profiles of the immune check points. Conversely, the injection of a population with a very low polyclonality increases the probability of T-cell exhaustion within tumor microenvironment, despite a high anti-tumor reactivity.

Considering this point, we analyzed the expression of a panel of activation/exhaustion markers through multiparametric flow cytometry on each infused T-cell population. We and others have previously suggested that PD-1 and TIGIT co-expression may define a relevant CD8 T cell population (thereafter called DPOS), of high functional avidity, associated with clinical efficacy of PD-1 blockade [22, 23, 29–31]. This DPOS subset is a heterogeneous population containing both activated and exhausted T cells, and the delicate balance between these two differentiation status is probably a key feature of therapeutic efficacy. As expected, specific T cells from P5 contained a significant proportion of DPOS T cells either for Melan-A or MELOE-1-specific T cells (Fig. 5a and b). This was also the case for specific T cells from P10 patient, but contrary to DPOS T cells from P5, these T cells contained a high proportion of CD39^+^, KLRG1^+^ and CD103^+^ T lymphocytes, suggesting an advanced exhaustion status. Indeed, it has been previously shown that CD39 and CD103 co-expression identifies a subpopulation of TILs found in tumor microenvironment, with a T_RM_ phenotype, expressing high levels of exhaustion markers, and enriched in tumor reactive T cells. Nonetheless, repetitive antigen stimulation led to impaired effector function and tumor escape [32]. Furthermore, the co-expression of KLRG1 on these specific T-cells also suggests an engagement into the exhaustion process [33].

In conclusion, we have demonstrated the feasibility of adoptive transfer of T lymphocytes from the endogenous repertoire of patients, produced according to an original method never tested before. We also demonstrated the safety of targeting the non-classical MELOE-1 antigen, never targeted before in immunotherapy protocols. Of the 6 patients treated, one patient developed a clinical response, and we hypothesize that this therapeutic response is related to the diversity and functional avidity of the Melan-A and MELOE-1 specific T lymphocytes that this patient received. This feature is strongly associated with the fraction of DPOS T cells and the activation/exhaustion status of antigen-specific repertoires.

This study demonstrates that the use of endogenous T lymphocytes, in addition to its safety, can lead to therapeutic efficacy, which can be further enhanced by the selection of highly reactive T cells, based on PD-1 and TIGIT co-expression. Such a therapy could be considered for patients refractory to ICI strategies, which would provide these patients directly with a highly tumor-specific T-repertoire, whose activity could be further enhanced by a combination with ICI.

## Supplementary Information

Below is the link to the electronic supplementary material.

Fig. S1: Distribution of non-serious events by SOC (System Organ Class). The coding of adverse events and reactions used the MedDRA thesaurus.Fig. S2: Functional avidities of the main TRBV subfamilies of Melan-A and MELOE-1-specific CTL lines. T cell functional avidities were evaluated by measuring CD107a mobilization in response to T2 cells loaded with a range of Melan-AA27L (upper panel) or MELOE-136-44 peptides, at an E:T ratio of 1:2. CD107a positive fraction was evaluated by flow cytometry with a specific antibody, gated on the relevant TRBV subtypes or on total CD8 T cells.Fig. S3: Gating strategy for phenotypic characterization of infused T-cells. Tetramer positive cells were first analyzed on viable cells, within singlets among lymphoid cells. Activation/exhaustion markers were then analyzed on tetramer-positive cells.Fig. S4 : Chemokine receptors expression by infused T-cells. The expression of various chemokine receptors was assessed on Melan-A and MELOE-1 specific T lymphocytes, through a multipanel labeling analyzed on a BD-Symphony cytometer. (PDF 466 kb)

Supplementary file2 (PDF 43 kb)

## Data Availability

The dataset used and/or analyzed during the current study is available from the corresponding authors on reasonable request.
