# Supplementary figures and images for: Phase I/II clinical trial of adoptive cell transfer of sorted specific T cells for metastatic melanoma patients
Source: Cancer Immunol Immunother. 2021 Jun 12;70(10):3015–30. doi: 10.1007/s00262-021-02961-0 (PMC8423703; doi:10.1007/s00262-021-02961-0)

**Fig S1**

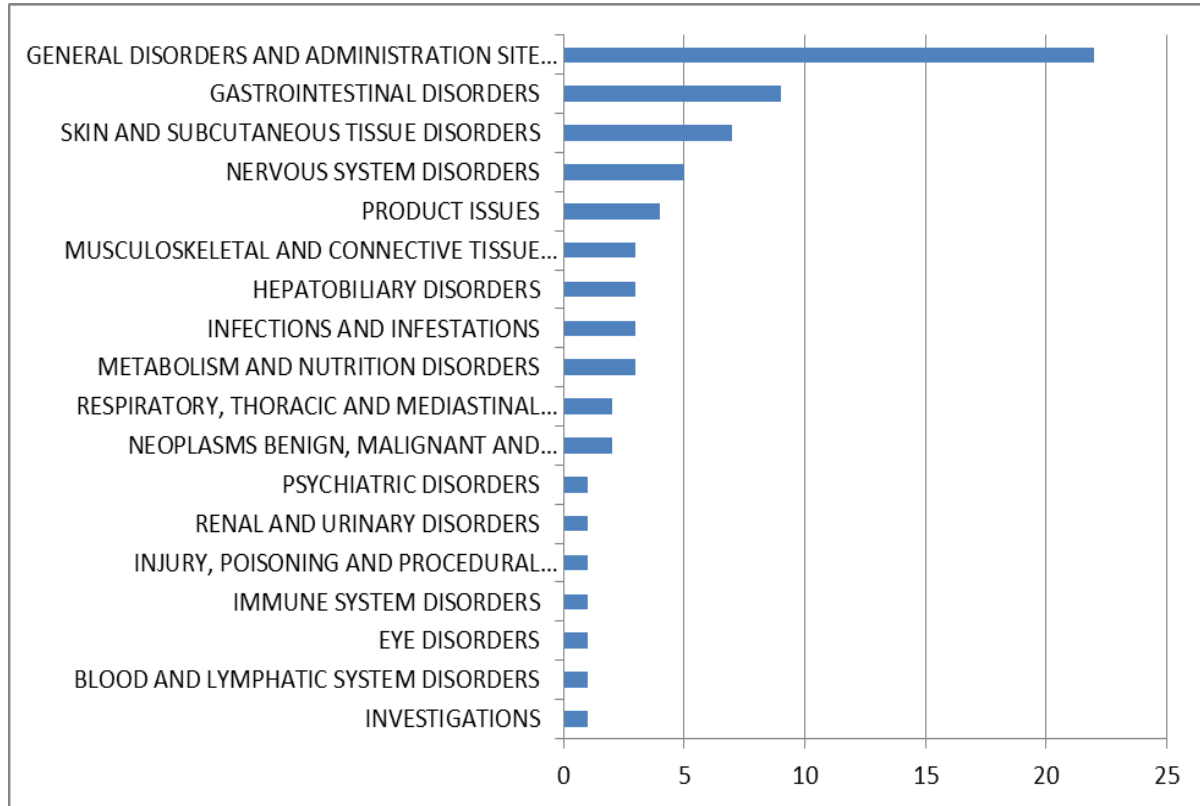

Fig S2

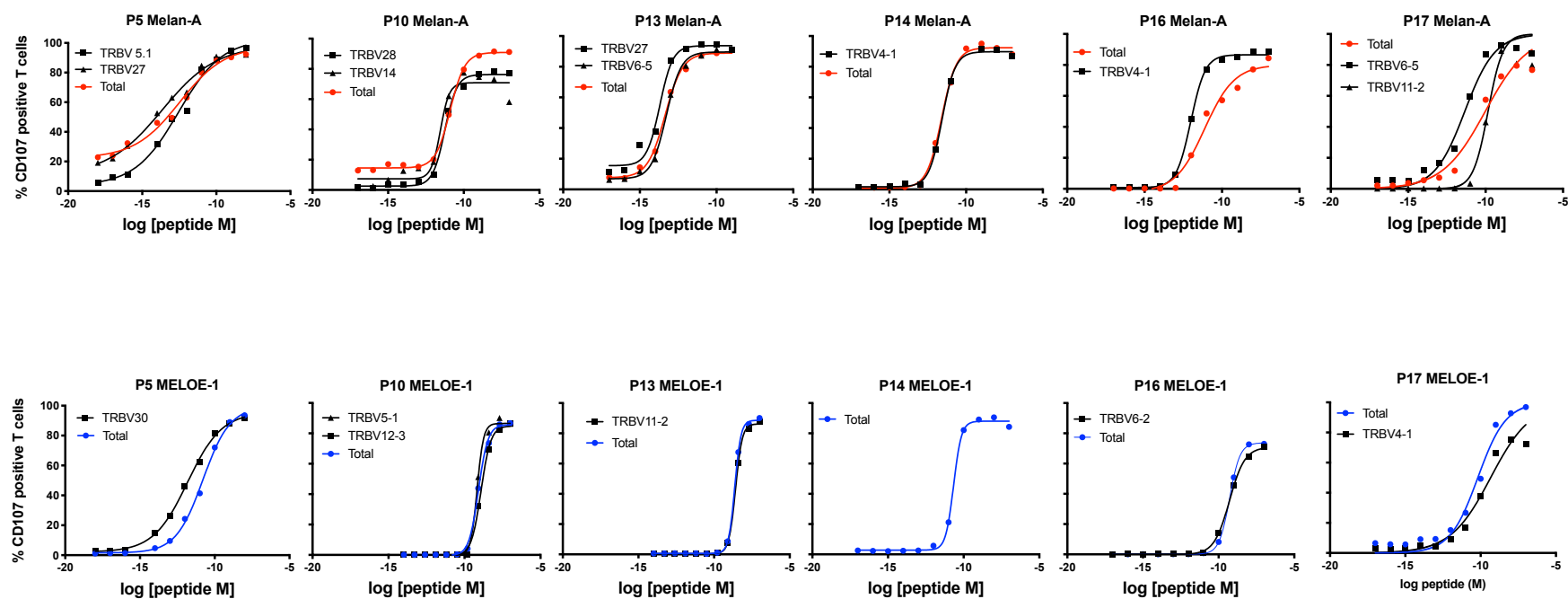

Fig S3

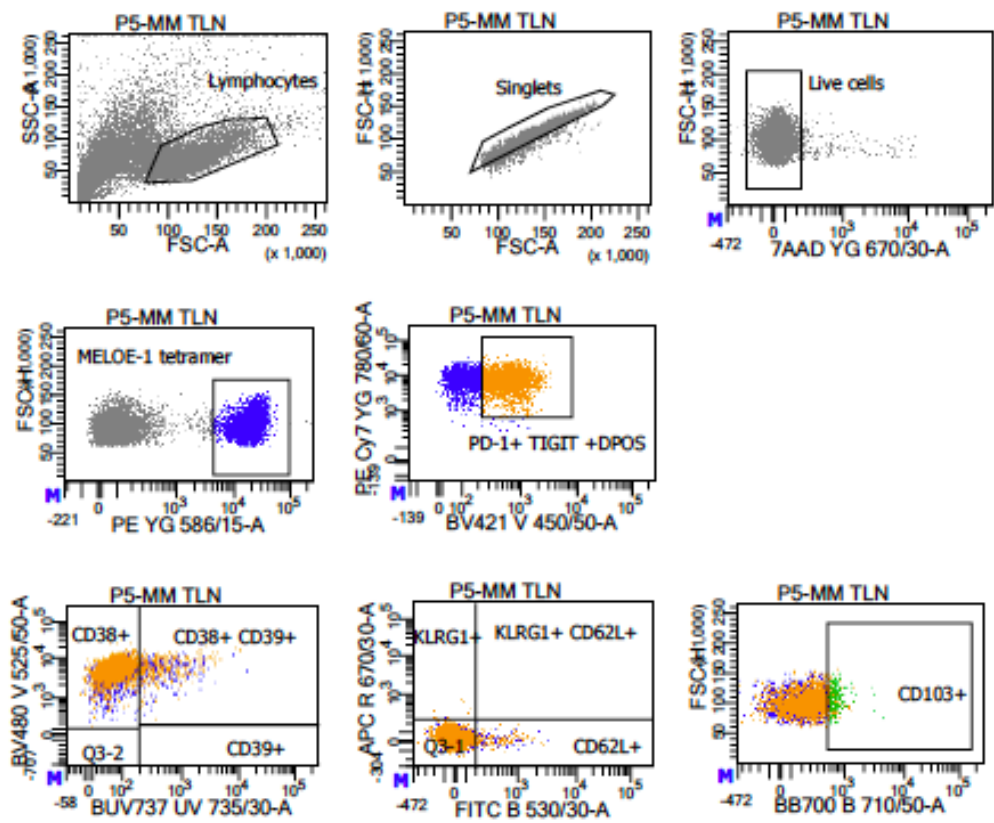

Fig S4

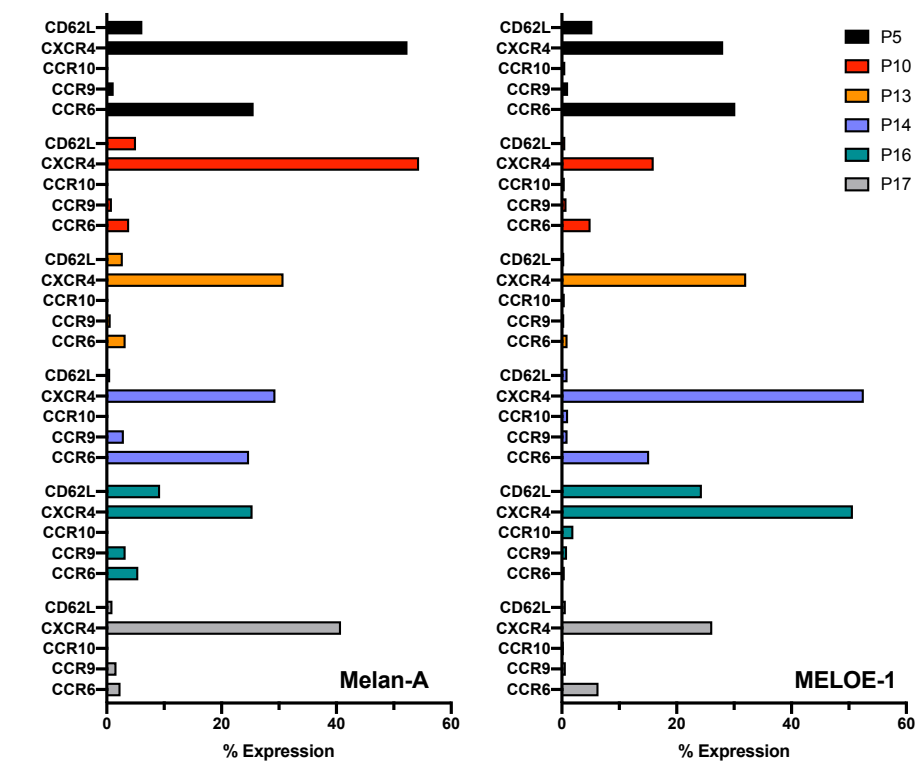

Supplement: Supplementary file 1 — Fig. S1: Distribution of non-serious events by SOC (System Organ Class). The coding of adverse events and reactions used the MedDRA thesaurus.Fig. S2: Functional avidities of the main TRBV subfamilies of Melan-A and MELOE-1-specific CTL lines. T cell functional avidities were evaluated by measuring CD107a mobilization in response to T2 cells loaded with a range of Melan-AA27L (upper panel) or MELOE-136-44 peptides, at an E:T ratio of 1:2. CD107a positive fraction was evaluated by flow cytometry with a specific antibody, gated on the relevant TRBV subtypes or on total CD8 T cells.Fig. S3: Gating strategy for phenotypic characterization of infused T-cells. Tetramer positive cells were first analyzed on viable cells, within singlets among lymphoid cells. Activation/exhaustion markers were then analyzed on tetramer-positive cells.Fig. S4 : Chemokine receptors expression by infused T-cells. The expression of various chemokine receptors was assessed on Melan-A and MELOE-1 specific T lymphocytes, through a multipanel labeling analyzed on a BD-Symphony cytometer. (PDF 466 kb) [file 262_2021_2961_MOESM1_ESM.pdf]
