# Supplementary material for: Phase I/II clinical trial of adoptive cell transfer of sorted specific T cells for metastatic melanoma patients
Source: Cancer Immunol Immunother. 2021 Jun 12;70(10):3015–30. doi: 10.1007/s00262-021-02961-0 (PMC8423703; doi:10.1007/s00262-021-02961-0)
Supplement: Supplementary file 2 — Supplementary file2 (PDF 43 kb) [file 262_2021_2961_MOESM2_ESM.pdf]

Table S1 : Patients included in the MELSORT trial

Patient number	*Birth date*	*Treatment*	*Remarks*
PM-05	07/02/1944	YES	
RC-10	30/03/1958	YES	
TV-13	30/04/1949	YES	
CO-14	11/12/1939	YES	
CM-16	26/09/1944	YES	
MF-17	15/01/1975	YES	
			Improper inclusion criteria[1]
PP-03	07/08/1943	NO	No HLA-A*0201 haplotype
CC-09	16/05/1961	NO	No HLA-A*0201 haplotype
GF-12	28/09/1939	NO	No HLA-A*0201 haplotype
MF-07	09/02/1952	NO	Lymphopenic patient : Lymphocytes < 1500 / µL
KP-19	14/10/1957	NO	Lymphopenic patient : Lymphocytes < 1500 / µL
LE-20	06/02/1952	NO	Lymphopenic patient : Lymphocytes < 1500 / µL
OA-08	10/04/1946	NO	No Melan-A expression in the tumor
			Defective T-cell production process[2]
SC-01	25/05/1946	NO	No Melan-A-specific T cells after the peptide stimulation step
BM-02	15/01/1964	NO	No Melan-A nor MELOE-1-specific T cells after the peptide stimulation step
SE-04	21/04/1961	5 x 10^8 Melan-A-specific CD8 T-cells	No MELOE-1-specific T cells after the peptide stimulation step<br>Compassionate treatment
CJ-06	18/03/1946	NO	No Melan-specific T-cells after the sorting step
PA-18	10/03/1942	NO	Insufficient number and reactivity of MELOE-1 specific T-cells at the end of the production process
			Other[3]
CM-11	29/08/1942	NO	Awaiting amendment approval from the ANSM: No authorization for inclusion
DM-15	08/05/1933	NO	General alteration of the patient status during the production process: transition to palatial care

[1]Pre-included patients withdrawn from the study due to improper inclusion criteria (no production process initiated). [2] Patients who did not receive the ACT treatment due to antigen-specific T cell repertoire deficiency, improper sorting yield or poor quality of expanded T-cells.[3] Pre-included patients withdrawn from the study for administrative issues or patient's health status (no production process initiated).

Table S2 : Blood frequencies of tetramer-positive T-lymphocytes among $CD8^+$ T cells

	Day 0		Day 1		Day 7		Day 30		Day 90	
	Melan-A	MELOE-1	Melan-A	MELOE-1	Melan-A	MELOE-1	Melan-A	MELOE-1	Melan-A	MELOE-1
P5 SM	$1.3x10^{-4}$	$1.8x10^{-4}$	$2.3x10^{-4}$	$2x10^{-4}$	$3.3x10^{-4}$	$4.7x10^{-4}$	$4.5x10^{-4}$	$1.4x10^{-4}$	$2.6x10^{-4}$	$4.6x10^{-4}$
P10 RC	$3.5x10^{-4}$	$1.3x10^{-4}$	$7.6x10^{-3}$	$7.3x10^{-3}$	$6.9x10^{-4}$	$3.9x10^{-4}$	$4.5x10^{-4}$	$1.1x10^{-4}$	$3.6x10^{-4}$	$1.3x10^{-4}$
P13 TV	$1.1x10^{-4}$	$1.7x10^{-4}$	$2.1x10^{-2}$	$1.9x10^{-2}$	$3.6x10^{-4}$	$7.6x10^{-4}$	$2.7x10^{-4}$	$1.2x10^{-4}$	$2x10^{-4}$	$1.5x10^{-4}$
P14 CO	$2.8x10^{-4}$	$5.5x10^{-4}$	$1.3x10^{-2}$	$3.4x10^{-3}$	$2.9x10^{-4}$	$6.7x10^{-4}$	$3.1x10^{-4}$	$4.4x10^{-4}$	$3x10^{-4}$	$2.1x10^{-4}$
P16 CM	$3.5x10^{-4}$	$2.3x10^{-4}$	$6.3x10^{-4}$	$3.1x10^{-4}$	$4.5x10^{-4}$	$4.5x10^{-4}$	$3.3x10^{-4}$	$2x10^{-4}$	$2.8x10^{-4}$	$1x10^{-4}$
P17 MF	$3.7x10^{-4}$	$8.9x10^{-5}$	$5.4x10^{-4}$	$2.1x10^{-4}$	$6.4x10^{-4}$	$2.1x10^{-4}$	$4.5x10^{-4}$	$1.3x10^{-4}$	ND	ND

Total blood (3 mL) from melanoma patients at different time-points, were incubated with 10 μg/mL of either Melan-A or MELOE-1/HLA-A*0201 APC-tetramers and with PE-conjugated anti-CD8 and FITC-conjugated anti-CD3, for 1h at 4°C in the dark, with gentle agitation every 15 minutes. After incubation, the red blood cells are lysed during 15 min at room temperature. After washing in PBS-0.1%BSA, cells were resuspended in PBS and analyzed on a FACSCanto. Frequencies of antigen-specific T-cells are calculated based on the number of tetramer positive cells, divided by the total number of $CD3^+/CD8^+$ T lymphocytes analyzed.
